# Kyste hydatique pancréatique: à propos d'un cas

**DOI:** 10.11604/pamj.2015.21.273.7267

**Published:** 2015-08-11

**Authors:** Rifki Saad Eljai, Rachid Boufettal, Robleh Hassan Farah, Farid Chehab

**Affiliations:** 1 Service de Chirurgie Viscérale Aile III, Centre Hospitalier Universitaire Ibn Rochd, Université Hassan II, Casablanca, Morocco

**Keywords:** Kyste hydatique, douleurs abddominales, pancréas, hydatid cyst, abdominal pain, pancreas

## Abstract

La localisation pancréatique du kyste hydatique est rare, même dans les pays ou la maladie hydatique sévit à l’état endémique. Il ne représentant que moins de 1% de l'ensemble des localisations.La symptomatologie souvent insidieuse après une longue évolution, dépend du siège du kyste hydatique, ce qui peut expliquer les difficultés du diagnostic, prêtant à confusion avec les autres lésions kystiques du pancréas. Elle touche exceptionnellement l'enfant. Nous rapportons une observation survenue chez une patiente de 20 ans, victime il y'a 3 ans d'un traumatisme fermé de l'abdomen, qui présentait depuis 2 mois des épigastralgies isolées, avec ictère. A travers cette observation et une revue de la littérature, nous discutons les difficultés diagnostiques et les modalités du traitement chirurgical de cette localisation inhabituelle de la maladie hydatique.

## Introduction

La localisation pancréatique du kyste hydatique est exceptionnelle [1–20]. Elle représente moins de 1% de l'ensemble des kystes hydatiques en zone d'endémie [1, 2]. Malgré l'apport de l'imagerie moderne, le diagnostic reste difficile. A travers cette observation, nous essayons de préciser les difficultés du diagnostic et de décrire les particularités du traitement chirurgical de cette affection.

## Patient et observation

**Figure 1:**
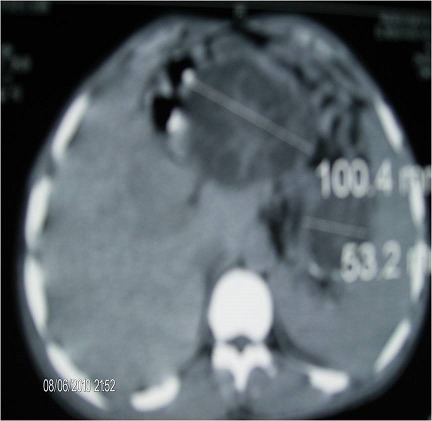
TDM abdominale montre une masse avec coque peu épaisse et une vésicule endokystique, avec un développement exophytique

**Figure 2:**
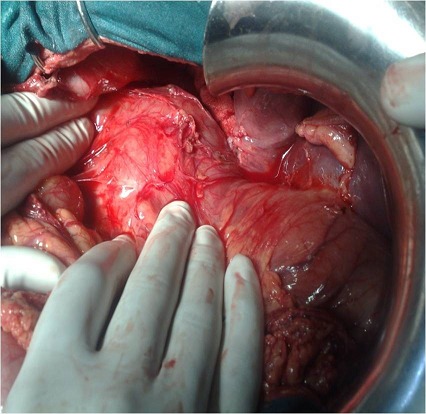
l'exploration trouvait un kyste hydatique corporéo-caudale à contenu uni-vésiculaire

**Figure 3:**
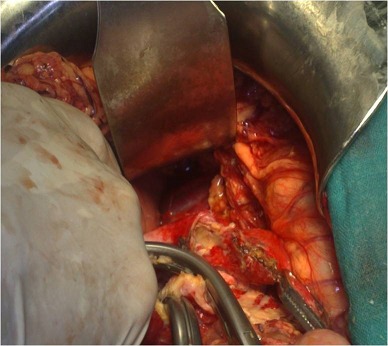
vue peroperatoire de la ponction vidange et une stérilisation du kyste, suivie d'une résection du dôme saillant et un drainage

Il s'agissait d'une patiente âgée de 20 ans, victime il y'a 3 ans d'un traumatisme fermé de l'abdomen et originaire d'une zone d'endémie hydatique. Elle était hospitalisée pour des douleurs épigastriques avec ictère, sans autre signe associé évoluant depuis 2 mois. L'examen clinique avait trouvé une sensibilité épigastrique sans masse palpable. Le bilan biologique montrait une cholestase (bilirubine totale a ‘ 140 mmol/L, bilirubine conjugue à 80 mmol/L, phosphatases alcalines à 700 UI/L) et une cytolyse (aspartate aminotransférase 320 UI/L, alanine aminotransferase 288 UI/L), le taux de prothrombine était à 75%. La fonction rénale était normale ainsi que l'amylasémie (50UI/L). L’échographie abdominale retrouvait, au niveau du pancréas dans sa partie corporéo-caudale, une formation lobulée anéchogène avec présence d'une vésicule endokystique, évoquant en premier lieu un cystadénome. La TDM abdominale, confirmait l'existence de cette masse avec coque peu épaisse et une vésicule endokystique, avec un développement exophytique Figure 1. Le CA 19-9 était normal, la lipasémie et la radio du thorax étaient normales. La sérologie hydatique n’était pas demandée.Le diagnostic d'une formation kystique du pancréas était retenu, mais sa nature hydatique n’était pas reconnue. La patiente était opérée par voie sous-costale gauche élargie à droite. L'exploration trouvait un kyste hydatique corporéo-caudale à contenu uni-vésiculaire Figure 2. Après protection par des champs imbibés d'une solution scoliocide, il a été réalisé une ponction vidange et une stérilisation du kyste, suivie d'une résection du dôme saillant et un drainage Figure 3. Les suites opératoires étaient simples avec une régression de l'ictère, une disparition de la douleur et une normalisation du bilan biologique. La patiente sortait au 10e jour postopératoire. Avec un recul de 22 mois, la patiente était asymptomatique. Les contrôles échographiques n'avaient pas décelé de récidive hydatique et l'immunologie était négative.

## Discussion

L'hydatidose est secondaire au développement chez l'homme de la forme larvaire de l'Echinococcus granulosus [1]. La localisation pancréatique représente moins de 1% des kystes hydatiques et 0,2% des localisations abdominales [2, 3]. Elle est isolée dans 91% des cas avec une légère prédilection pour la portion céphalique (57%) [2, 4]. Le kyste hydatique du pancréas (KHP) n'a été qu'exceptionnellement rapporté chez l'enfant [4, 9]. Le mode d'infestation du pancréas se fait par voie hématogène après passage des filtres hépatique et pulmonaire [1]. Au niveau du pancréas, le kyste augmente progressivement de volume, refoule le parenchyme pancréatique, comprime puis érode les organes avoisinants. Dans notre observation, le kyste comprimait le second duodénum et les canaux biliaires. Le KHP n'a pas de signes cliniques spécifiques. Sa symptomatologie est fonction du siège et de la taille du kyste [1, 4]. Cette localisation inhabituelle peut en effet être révélée par une douleur épigastrique chronique, un ictère rétentionnel (localisation céphalique) ou une masse épigastrique [5–7, 9, 11]. Dans notre observation, le signe révélateur était une douleur épigastrique. Mais le plus souvent, le diagnostic se fait après complication du kyste: suppuration [4], fistulisation dans la voie biliaire principale [4], hypertension portale par compression de la veine splénique, pancréatite aiguë [12, 13]. L’échographie, la tomodensitométrie et l'imagerie par résonance magnétique (IRM), permettent sans difficulté de retenir le diagnostic d'une lésion kystique pancréatique [9, 14, 15, 20], mais le diagnostic préopératoire de la nature hydatique du kyste est extrêmement difficile à reconnaître [7, 9, 20]. Néanmoins, certains signes peuvent aider à évoquer le diagnostic notamment des calcifications périkystiques, la présence de vésicules intrakystiques, un décollement de la membrane hydatique ou l'association d'autres localisations plus évidentes de kyste hydatique (foie) [14]. En cas de persistance d'un doute diagnostic, le recours à l’écho-endoscopie est d'un grand apport car elle permet une meilleure étude du contenu kystique [16]. Cependant, la confrontation des données épidémiologiques, radiologiques (échographie, tomodensitométrie, IRM et éventuellement, écho-endoscopie) et immunologiques permettent, parfois, de confirmer la nature hydatique d'une masse kystique pancréatique [3, 5, 6, 11, 17]. Le diagnostic différentiel se pose avec les autres tumeurs macro-kystiques du pancréas [18, 20]. Le pseudo-kyste se distingue de la maladie hydatique par l'absence d'une paroi propre, l'histoire et le contexte cliniques. Le cystadénome et le cystadénocarcinome se caractérisent par le rehaussement, après injection du produit de contraste au scanner, des bords et des cloisons intra-kystiques [18]. Le traitement du KHP est chirurgical [19]. Le choix du geste dépend du siège du kyste et de l'existence ou non d'une fistule kystocanalaire [4]. En fait, il est actuellement admis par la majorité des auteurs que pour les localisations corporéocaudales, la morbidité du drainage après résection du dôme saillant (fistule pancréatique) doit faire préférer les interventions d'exérèse type spléno-pancréatectomie gauche [17, 20]. En revanche, pour les kystes céphaliques, le traitement de référence est une résection du dôme saillant associée, en cas de fistule canalaire, à une anastomose kysto-digestive [11]. Cette dérivation de type anastomose kysto-gastrique ou kysto-duodénale, ou kysto-jéjunale sur anse en Y doit être préférée au drainage externe ou à l'unique résection du dôme saillant avec ou sans épiploplastie du fait de la morbidité qui en résulte [10, 20]. Ce geste peut être difficile et dangereux si le parenchyme pancréatique est friable. Dans ce cas, une suture canalaire sur un drain tuteur pourrait être envisagée. Dans notre observation, nous avons réalisé d'une résection du dôme saillant et un drainage. La duodénopancréatectomie céphalique constitue un geste radical mais semble démesurée pour une pathologie bénigne [4]. La compression de la voie biliaire principale régresse après traitement du kyste et ne nécessite aucun geste sur le cholédoque [4].

## Conclusion

La localisation pancréatique primitive du kyste hydatique est exceptionnelle. L'origine hydatique doit être évoquée devant toute masse kystique de la région céphalique du pancréas survenant chez un enfant présentant un ictère cholestatique et provenant d'une zone d'endémie. Le traitement chirurgical doit être dans la mesure du possible conservateur en réalisant des dérivations kysto-digestives afin de prévenir les fistules pancréatiques [20].
